# Toward transcriptomics as a primary tool for rare disease investigation

**DOI:** 10.1101/mcs.a006198

**Published:** 2022-02

**Authors:** Stephen B. Montgomery, Jonathan A. Bernstein, Matthew T. Wheeler

**Affiliations:** 1 Departments of Genetics and Pathology, Stanford University, California 94305, USA;; 2 Department of Pediatrics, Stanford University School of Medicine, Stanford, California 94305, USA;; 3 Division of Cardiovascular Medicine, Department of Medicine, Stanford University, California 94304, USA

## Abstract

In the past 5 years transcriptome or RNA-sequencing (RNA-seq) has steadily emerged as a complementary assay for rare disease diagnosis and discovery. In this perspective, we summarize several recent developments and challenges in the use of RNA-seq for rare disease investigation. Using an accessible patient sample, such as blood, skin, or muscle, RNA-seq enables the assay of expressed RNA transcripts. Analysis of RNA-seq allows the identification of aberrant or outlier gene expression and alternative splicing as functional evidence to support rare disease study and diagnosis. Further, many types of variant effects can be profiled beyond coding variants, as the consequences of noncoding variants that impact gene expression and splicing can be directly observed. This is particularly apparent for structural variants that disproportionately underlie outlier gene expression and for splicing variants in which RNA-seq can both measure aberrant canonical splicing and detect deep intronic effects. However, a major potential limitation of RNA-seq in rare disease investigation is the developmental and cell type specificity of gene expression as a pathogenic variant's effect may be limited to a specific spatiotemporal context and access to a patient's tissue sample from the relevant tissue and timing of disease expression may not be possible. We speculate that as advances in computational methods and emerging experimental techniques overcome both developmental and cell type specificity, there will be broadening use of RNA sequencing and multiomics in rare disease diagnosis and delivery of precision health.

## MEASURING RARE VARIANT EFFECTS USING TRANSCRIPTOMICS

Transcriptome or RNA-sequencing (RNA-seq) in specific cell types or tissues can provide robust quantification for the expression levels of more than 8000 genes and further tens of thousands of splice junctions, providing extensive coverage of a broad range of molecular events. In contrast to targeted methods of RNA quantification such as reverse transcription polymerase chain reaction (RT-PCR), this approach can provide a broad view of transcription-related molecular events. In addition, the types of molecular events evaluated by RNA-seq extend beyond expression and splicing levels of known gene products as identification and quantification of noncoding genes, novel transcripts, fusion genes, retained introns, alternative polyadenylation, and transcription starts can be determined. Further, additional molecular signals are also quantifiable including allele-specific expression, nonsense-mediated decay, and RNA editing.

Each transcript measured by RNA-seq is subsequently amenable to genetic analyses. When profiled in human population samples, genetic association analyses have uncovered abundant quantitative trait loci (QTLs) for common genetic variants (The GTEx Consortium 2020). However, extending these analyses to rare variant effects has been more challenging because of increased uncertainty around effect sizes and the abundance of rare variants to test in any human population sample (Keinan and Clark 2012; Bomba et al. 2017). To mitigate some of these challenges and focus on impactful rare variants, analyses can be restricted to rare variants associated with outlier expression measurements. As such, we and others have shown that genes with outlier expression in an individual are enriched in gene proximal rare variants (Montgomery et al. 2011; Zeng et al. 2015; Zhao et al. 2016; Li et al. 2017). These enrichments were further significant for all major classes of genetic variants but were most striking for rare structural variants reinforcing the ongoing needs for accurately identifying structural variants in individual genomes and the use of transcriptomes to guide interpreting variant effects (Ferraro et al. 2020). Furthermore, these enrichments informed both protein-coding and noncoding variant effects; rare, protein-truncating variants were highly enriched in individuals with outlier gene expression because of the effects of nonsense-mediated decay on gene expression, and rare, predicted splicing variants were highly enriched in individuals with outlier splicing levels. To continue the discovery of outlier-associated rare variants will require that future transcriptome studies are complemented by high-quality whole-genome data. Further, as these studies have focused predominantly on expression and splicing outliers, other outlier molecular effects from the range of multiomics assays are only beginning to be systematically integrated with transcriptome data (Kopajtich et al. 2021).

When considering the potential for transcriptome sequencing for rare disease diagnosis, these studies in healthy individuals have demonstrated that outlier effects can be driven by diverse categories of rare variants. They have demonstrated that RNA-seq can facilitate detecting the effects of specific candidate variants, particularly for splicing. Further, they have demonstrated that computational methods that combine both whole-genome and RNA-seq data from the same individual can enhance the prioritization of large-effect, rare variants and such variants can have increased impacts on diverse traits and diseases (Li et al. 2017; Ferraro et al. 2020). However, there are few other computational methods that integrate individual genomes and transcriptomes to prioritize rare variants, particularly in comparison to variant effect prediction tools that use genome data alone. This is an area of method development that will grow as future approaches combine advances in machine learning for variant effect prediction with individual -omics data to improve discovery of pathogenic rare variants.

## DIAGNOSTIC YIELD OF TRANSCRIPTOMICS IN RARE DISEASES

It is estimated that the current diagnostic yield of DNA sequencing is 25%–30% in large, heterogeneous rare disease cohorts (Jacob et al. 2013; Yang et al. 2013; Iglesias et al. 2014; Lee et al. 2014; Yang et al. 2014; Posey et al. 2016; Deciphering Developmental Disorders Study 2017; Tan et al. 2017; Clark et al. 2018). In homogeneous rare diseases cohorts, the diagnostic yield can range from 40% to 60% (Shashi et al. 2014; Ankala et al. 2015; Wortmann et al. 2015; Yuen et al. 2015; Splinter et al. 2018; 100,000 Genomes Project Pilot Investigators et al. 2021). To investigate further increases to this yield, a number of studies in the past 5 years have used RNA sequencing (Cummings et al. 2017; Kremer et al. 2017; Frésard et al. 2019; Gonorazky et al. 2019; Lee et al. 2020; Rentas et al. 2020; Murdock et al. 2021; Yépez et al. 2021). The public availability of RNA-seq data from rare disease cohorts is poised to accelerate; for example, the Undiagnosed Disease Network has shared RNA-seq data for 816 patient samples (phs001232.v4.p2) and the National Institutes of Health (NIH) Centers for Mendelian Genomics Consortium has generated RNA-seq data for 714 patient samples (Baxter et al. 2022). Across these research studies, the use of transcriptomics has predominantly been a secondary activity aimed to evaluate if aberrant expression or splicing events can improve identification of pathogenic variants in patients in whom DNA sequencing alone has not yielded a definitive genetic cause. Despite recent advances, clinical transcriptomic testing for Mendelian conditions is currently available from only a few reference laboratories (https://www.ncbi.nlm.nih.gov/gtr/).

The earliest application of transcriptomics to enhance diagnostic yield in a DNA-sequenced rare disease cohort was conducted by Cummings et al. (2017) to survey 50 patients with genetically undiagnosed rare muscle disorders. Cummings et al. focused on analysis of muscle tissue in comparison to 180 healthy controls muscle samples from the NIH Common Fund Genotype Tissue Expression (GTEx; gtexportal.org) project to identify predominantly splice-altering variants and reported a diagnostic yield of 35%. Kremer et al. (2017) conducted another study in which they generated fibroblast transcriptomes for 48 patients with undiagnosed rare mitochondriopathies and compared them to a cohort of 105 patients (including the original 48) to achieve a diagnostic yield of 10%. Our own work evaluated the utility of blood transcriptomes for diverse rare disease cases encountered by a clinical genomics service (Frésard et al. 2019). We generated blood transcriptomes for a heterogenous rare disease cohort of 94 patients and compared them to nearly 1000 healthy control samples to achieve an incremental diagnostic yield of 7.5%. We further estimated the need for hundreds of healthy individual samples per tissue type to achieve robust estimates of a patient's outlier effect as increasing enrichments of outliers in loss-of-function intolerant genes were observed as a function of healthy reference sample size. Since these studies, a number of comparable analyses have systematically used RNA-seq to supplement rare disease investigations reporting increases in diagnostic yields ranging from 7% to 36% (Gonorazky et al. 2019; Lee et al. 2020; Rentas et al. 2020; Murdock et al. 2021; Yépez et al. 2021), and a growing number of reviews provide additional insights into this area of research (Saeidian et al. 2020; Lord and Baralle 2021; Macken et al. 2021; Ergin et al. 2022).

Despite the new diagnostic opportunities presented by these studies, they have also presented different diagnostic yields and collectively indicated several key factors when using transcriptome sequencing in a rare disease setting. A primary factor is the extent of prior information on both the mode of inheritance and likely causal genes. Relatively easier cases to diagnose using transcriptome sequencing are recessive diseases with known causal gene(s) and a yet-to-be-annotated pathogenic variant. Here, transcriptome sequencing can evaluate for splice or regulatory effects within the focus of the causal gene or gene set. This is further facilitated if one of the pathogenic alleles is already identified and the underlying cause is suspected to involve compound heterozygosity with the second, yet-to-be-discovered allele. At least three of the solved cases from Cummings et al. involved compound heterozygosity of a protein-coding and splicing variant. The most challenging cases to supplement with transcriptome sequencing remain somatic mosaic disorders (Ayturk et al. 2016) and disorders in which candidate genes remain unknown and few, if any, clinical diagnoses have been made.

Another critical factor in assessing the incremental diagnostic yield of RNA-seq in rare disease cohorts was study design. Unlike genome sequencing (NICUSeq Study Group et al. 2021), there has yet to be a randomized clinical trial on the diagnostic yield for RNA-seq. As such, existing studies are either exploratory or retrospective and can vary on how they define solved cases. The study design in Cummings et al. focused on rare myopathies using muscle RNA-seq, and ultimately 15 of their 17 novel diagnoses were within four well-known myopathy genes: *COL6A1*, *DMD*, *NEB*, and *TTN*. Cases were diagnosed if a complete genetic diagnosis could be inferred in the context of an outlier splice event. Kremer et al.’s study design focused on rare mitochondriopathies using fibroblast RNA-seq. The authors were able to report a novel disease association for *TIMMDC1* and considered cases solved if a disease-associated variant was detected and RNA-seq effects were validated in proteomics and/or biochemical assays. Fresard et al.’s study design focused on a heterogeneous mix of rare diseases using blood RNA-seq and was limited to defining a case solved only when clinically curated variants and outlier genes converged and a complete genetic explanation was possible. For each study, future refinements of candidate gene sets and follow-up validation experiments are expected to increase the diagnostic yield. To this point, both Kremer et al. and Fresard et al. indicated a high number of promising expression and splicing outlier genes in which a complete genetic diagnosis was yet to be confirmed.

## IMPACTS OF DEVELOPMENTAL AND CELL TYPE SPECIFICITY

An often-discussed challenge with use of transcriptome sequencing to aid rare disease diagnosis is the unknown impact of developmental and cell type specificity of gene expression. Several genetic diseases can occur because of mutations in tissue restricted transcripts in difficult to access tissues (i.e., cerebral cortex, myocardium). Multiple congenital disorders are already known to have developmental- and cell type–specific etiologies driven by specific enhancer mutations (Claringbould and Zaugg 2021). Existing transcriptome-based rare disease studies have profiled a range of relatively accessible tissues and cell lines from blood, fibroblasts, lymphoblastoid cell lines, or muscle. However, if a disease-causing rare variant's impact is restricted in time and space, profiling a more readily sampled cell type could fail to provide any meaningful extra diagnostic information and add additional patient burden and cost.

Assessing the extent and impact of cell type specificity of genetic effects was a major rationale for the GTEx project's survey of gene expression and splicing across the human body (The GTEx Consortium 2020). GTEx identified a U-shaped pattern for specificity of common variant effects with gene regulatory effects being either highly shared or highly tissue-restricted. GTEx also identified that a variant's proximity to a gene and a gene's expression level were good indicators of whether an effect would be observed in an unassayed tissue. The observation is expected to extend to rare variants as our own GTEx-based analysis of the expression level impacts of protein-truncating variants that induce nonsense-mediated decay exhibited minimal tissue variability, indicating that as long as the gene is expressed, transcriptome data can guide interpretation of multiple gene proximal variant effects (Teran et al. 2021).

For future rare disease studies, approaches that help identify the most informative cell types to study will also significantly aid in informed use of transcriptome data. We have recently seen the increased emergence of studies that demonstrate the utility of accessible cell types when the pathological tissue is hard to obtain. For example, Rentas et al. showed that lymphoblastoid cell lines derived from blood were highly relevant for neurodevelopmental rare diseases exhibiting broad isoform sharing with brain tissues and an overall ability to survey more than 1700 rare neurodevelopmental disease genes (Rentas et al. 2020). Complementing this are new tools that use phenotype information to guide the tissue-selection decision-making process. Velluva et al. (2021) recently reported the Phenotype-Tissue Expression and Exploration (PTEE) tool to guide selection of analysis tissues in different disease contexts. Future approaches may also begin to triangulate a subset of assayable cell types that are most relevant for a patient instead of selecting a single tissue to analyze. In our own work, we observed that combining undifferentiated induced pluripotent stem cells (iPSCs) and blood transcriptomes from the same patient could enhance detection of outlier events and rapidly narrow candidate disease genes (Bonder et al. 2021). Likewise, Murdock et al. (2021) studied outliers in both blood and fibroblasts from the same patients.

Beyond bulk transcriptomes, single-cell multiomics offers multiple emerging opportunities to map developmental and cell type–specific effects. Large-scale single-cell catalogs such as the Human Cell Atlas and Tabula Sapiens provide opportunities to map expression levels of candidate genes in highly specific cell types across the human body (Regev et al. 2017; The Tabula Sapiens Consortium and Quake 2021). Future bulk and single-cell data sets from the Developmental GTEx (dGTEx) project will increase our understanding of human population variability in gene expression and splicing through development. Further, reference single-cell chromatin accessibility maps provide annotation of developmental and cell type–specific regulatory elements as potential targets of rare variant effects. The utility of these data is apparent in a recent map of single-cell chromatin accessibility in the developing cerebral cortex that identified an enrichment of de novo mutations from autism cases in the accessible chromatin of developing radial glial cells—an enrichment that authors noted was comparable to deleterious protein-coding mutations (Trevino et al. 2021). These reference single-cell data, when combined with advances in noncoding variant effect prediction, will increasingly aid the prediction of development and cell type–specific pathological contexts and the experimental cellular proxies or analysis tissues for functional validation assays of patient-derived variants and mutations.

## COMPUTATIONAL METHODS FOR TRANSCRIPTOMICS IN RARE DISEASE

Recent use of transcriptomics in rare diseases has led to multiple computational advances. One class of such advances has been pipelines and tools to define robust outlier gene expression or splicing events. Most prior studies have used a combination of defining *z*-score thresholds and/or assessed differential expression with DESeq2 (Love et al. 2014). This approach requires careful normalization of control data with respect to case data as there are often few rare disease samples sequenced separately from the majority of reference healthy controls. One approach to overcoming this has been to model and regress out known and latent factors across sequencing batches (Frésard et al. 2019). Separate computational tools called OUTRIDER and FRASER have been developed specifically for the task of rare disease diagnosis with RNA-seq by providing an end-to-end approach for correcting technical noise and providing a statistical test for expression and splicing outliers (Brechtmann et al. 2018; Mertes et al. 2021). These tools model latent factors using an autoencoder and report statistical significance from a negative-binomial or β-binomial distribution. For splicing alone, the LeafCutterMD tool has provided an approach to detecting outliers and was designed to overcome class imbalance issues present when comparing small numbers of patient samples to multiple controls (Jenkinson et al. 2020). For each outlier detection approach, once a patient's transcriptome sample has been processed, there remains the possibility that too many outliers are detected because of sample-specific issues that corrupt the measurement of many genes. To address this, we have often removed or reprocessed samples with abundant outliers (i.e., >50). Additionally, defining a meaningful outlier threshold for investigation can vary across studies and samples. In the cases in which there is a single known candidate gene, the threshold for outlier effects may benefit from being reduced or in some cases manually inspected (Lee et al. 2020).

Another computational approach for guiding identification of causal genes and variants in rare disease transcriptomes has relied on allele-specific expression (ASE) analysis. ASE is measurable by assessing the relative abundance of RNA sequencing read ratios over heterozygous coding variant sites; however, such variants need not be causal themselves (Castel et al. 2015). Several previously mentioned transcriptome studies from rare disease cohorts have used ASE as an additional signal to inform outlier events or potential haploinsufficiency. An additional study has also demonstrated more generally how ASE can identify likely Mendelian disease genes in which protein-truncating variants escape nonsense-mediated decay (Coban-Akdemir et al. 2018). To calculate ASE, most studies have used the WASP pipeline, which aims to overcome mapping biases for non–reference allele–containing sequencing reads (Geijn et al. 2015). For rare disease transcriptome analysis, the computational tool ANEVA-DOT has further provided an approach to identify outlier ASE (Mohammadi et al. 2019). When applied to cases from Cummings et al., 76% of cases had outlier ASE in a confirmed disease gene. Notably, this was among a small number of ASE outlier events detected per individual. Future computational approaches can likely increase the use of ASE in rare disease settings by integrating outlier expression and allele-specific expression into a combined outlier detection model.

Supplementing exome data, methods for calling variants from transcriptome data provide an opportunity for identifying rare coding alleles, splice sites, and structural variants. One such tool, MINTIE, was applied to data from Cummings et al. and showed detection of 9 of 13 novel splice variants from 10 individuals and the identification of a previously unobserved fusion product in the *DMD* gene (Cmero et al. 2021). Both Gonorazky and Yépez et al. further have applied GATK-based RNA-seq variant calling in their rare disease cohorts to extend coverage and identification of pathogenic variants into untranslated regions (UTRs) (Gonorazky et al. 2019; Yépez et al. 2021; RNAseq Short Variant Discovery (SNPs+Indels)).

Integrative computational approaches that combine a patient's genome and transcriptome also provide exciting promise for aiding rare disease studies. This remains an area of nascent activity, and most rare disease transcriptomics studies often assess candidate variants near outliers manually. To improve this, the computational tools RIVER and WATERSHED jointly model transcriptome outliers and genomic annotation of proximal variants to prioritize likely causal rare variants (Li et al. 2017; Ferraro et al. 2020). WATERSHED provides the opportunity to jointly assess gene expression, splicing, and allele-specific expression outliers. These methods when combined with additional patient multiomics are likely to continue to enhance prioritization of impactful rare variants. A key to this ongoing method development will be data sharing of whole-genome, multiomics, and phenotype information from rare disease patients.

## ELIMINATING BARRIERS TO TRANSCRIPTOMICS IN RARE DISEASE STUDY AND DIAGNOSIS

Experimental advances over the last decade suggest that we are approaching a transition point at which diagnostic use of transcriptomes may overcome current limitations related to developmental and cell type specificity. Already skin biopsy–derived fibroblasts and peripheral blood–derived and –transformed lymphoblastoid cell lines are used as source materials in rare disease transcriptomic studies. Gonorazky et al. (2019) took this one step further by transdifferentiating fibroblasts to myotubes and generating RNA-seq to study the diagnostic yield for patients with undiagnosed neuromuscular disorders. Undifferentiated iPSC transcriptomes have demonstrated potential use for rare disease diagnoses by identifying disease-relevant outliers in a heterogeneous cohort of 65 rare disease patients (Bonder et al. 2021). As additional trans-differentiation and differentiation protocols become widely available and reproducible, the generation of cell types of interest for transcriptomics can allow interrogation of a larger portion of the “whole human transcriptome.” Just as genome sequencing has provided incremental diagnostic power versus exome sequencing, we believe interrogation of multiple cell types through transcriptomic profiling may be an effective approach to enhancing diagnostic yield in rare disease. Further, the rapidly emerging availability of organoid systems and embryoid bodies to explore a range of developmental and cell type contexts expands the opportunities to study outlier effects in patients (Rhodes et al. 2022).

The availability of these cellular models is also providing new opportunities to rapidly test the impacts of genomic variants in previously hard-to-access contexts. Use of massively parallel reporter assays (MPRAs) has enabled testing thousands of variants for regulatory function in multiple primary cell types (White 2015). Splicing and 3′-UTR variant MPRAs further provide opportunities to explore new classes of variant effects (Rosenberg et al. 2015; Griesemer et al. 2021). In parallel, high-throughput and multiplex CRISPRi and CRISPRa assays also provide the opportunity to inhibit and activate key regulatory regions harboring candidate variants and further provide new therapeutic opportunities to overcome haploinsufficiency in specific developmental and cell type contexts (Matharu et al. 2019). Strategies to test variant effects on gene expression and splicing in a range of different developmental and cell type contexts are already here.

With these advances it becomes possible to envision specific scenarios in which a patient's phenotype with, or even without, their genotype can indicate a range of cellular contexts to study and that the presence of an aberrant molecular event will be sufficient to nominate a causal gene. In some cases, knowing the specific causal variant may be secondary to a diagnosis, as the nature of an aberrant molecular event that integrates both the unseen rare variant and a patient's genetic background may be sufficient to support a diagnosis. There will of course be limitations as pathogenic variant effects that do not manifest on gene expression or downstream gene regulatory networks in any cellular context can be missed. Continued progress toward unlocking transcriptomics as a primary diagnostic tool at scale will require comprehensive maps of human cell types and their regulatory regions, activities already well underway. It will also require rapid and cost-effective, multiplexed cell culture, genetic or chemical perturbation assays that reexpose variant effects. Finally, it will require data sharing of reference population-scale databases to identify the normal ranges of molecular activity. With these resources, the future may see patients’ care informed by knowledge of their genetic variants and additionally their personal transcriptomic profile.

## ADDITIONAL INFORMATION

### Acknowledgments

S.B.M., J.A.B., and M.T.W. are supported by NIH grants U01HG010218 (UDN) and U01HG011762 (GREGoR). The content is solely the responsiblity of the authors and does not necessarily represent the official views of the National Institutes of Health. S.B.M. is a consultant for BioMarin Pharmaceutical, Myome Inc., and Tenaya Therapeutics.

### Competing Interest Statement

The authors have declared no competing interest.

### Referees

Julien Gagneur

Anonymous
